# From the perspective of multi-theory model, factors influencing physical activity among community-dwelling older adults with type 2 diabetes in China: a mixed-methods study

**DOI:** 10.3389/fpubh.2025.1634294

**Published:** 2025-09-25

**Authors:** Panpan Huai, Bo Zhang, Jingjing Sun, Rui Xu, Linghui Zhang, Xiao Qiao, Weili Sun, Hui Yang, Jinli Guo, Huancheng Su

**Affiliations:** ^1^School of Nursing, Shanxi Medical University, Taiyuan, China; ^2^Balingqiao Community Health Service Center of Xinghualing District, Taiyuan, China; ^3^Shanxi Bethune Hospital, Shanxi Academy of Medical Sciences, The Third Hospital of Shanxi Medical University, Tongji Shanxi Hospital, Taiyuan, China; ^4^The First Clinical Medical College of Shanxi Medical University, Taiyuan, China; ^5^The Second Clinical Medical College of Shanxi Medical University, Taiyuan, China

**Keywords:** multi-theory model, type 2 diabetes, older adult, community, factors, mixed-method study

## Abstract

**Aim:**

To examine the factors influencing physical activity among community-dwelling older adults with type 2 diabetes in China, and to provide a strong theoretical framework and empirical support for creating more individualized and scientific strategies for improving physical activity.

**Methods:**

The text was analyzed by the innovative combination of traditional thematic analysis method and topic modeling (python machine learning) through the qualitative study, aiming to deeply explore the experiences and views of older adults with type 2 diabetes in the community regarding physical activity. Quantitative study adopted a cross-sectional survey to objectively and efficiently discover causal relationships among data. Finally, the results from the two different researches were compared to identify differences, similarities, and contradictions, enabling mutual verification and supplementation of the research findings and compensating for the limitations of a single research method, thereby obtaining the ultimate results.

**Results:**

We compared and integrated the qualitative and quantitative study results, and finally determined 11 topics, including 23 key factors, as the set of the study results of this study.

**Conclusion:**

From the perspective of Multi-theory model, this study explored the related factors influencing the physical activity of older adults with type 2 diabetes in the community through a mixed-methods study. By comparing and integrating the results of qualitative and quantitative studies, we finally identified 11 topics, including 23 key factors, as the results of this study, such as “Focus on physical activity itself,” “The persistence of physical activity,” and “Traditional conception,” which were not mentioned in previous systematic literature search. These new discoveries provide a empirical support for creating more individualized and scientific strategies for improving physical activity behaviors among older adults with type 2 diabetes in the community.

## 1 Introduction

The *IDF Diabetes Atlas* shows (1) that there were about 500 million diabetes patients worldwide in 2021. It is projected that this number may increase to over 600 million by 2030. Among them, Type 2 diabetes (T2DM) is the most common type which is accounting for over 96% of all this disease throughout the world (2). The threat posed by T2DM to global health is increasing day by day. T2DM has a high disability rate and is one of the main causes of blindness and non-traumatic amputation (3), seriously damaging the quality of life of patients (4). Research data from *The Lancet* shows that by 2022, there were 148 million diabetes patients in China, accounting for 18% of the total number of diabetes patients all over the world. It is predicted that this number will increase to 175 million by 2045, ranking second globally (5). Among them, Older adults have the highest prevalence of T2DM of any age group and at least 20% of patients over the age of 60 have diabetes (more than 95% are T2DM) (6). Thanks to the improvement of health and medical conditions, the increase in educational opportunities and the decline in fertility rates, the global trend of population aging is accelerating, and population aging has become one of the key demographic changes faced by many countries (7). Diabetes, as one of the most common types of chronic diseases in older adults, has become a key factor hindering the improvement of health levels (8, 9). The lifestyle of patients and their self-management level play an important role in their health (10). The *American College of Lifestyle Medicine* regards lifestyle interventions as first-line management for diabetes (11). Physical activity, which is an essential part of behavior and lifestyle intervention (12), urgently needs to be given key attention.

In 2021, the *Association of Diabetes Care and Education Specialists* (ADCES) proposed ADCES7 Self-Care Behaviors, in order to assist people with diabetes in making effective changes to their self-management behaviors (13). Among them, Being active is located in the inner ring of the transformation of the ADCES7 image and is served as the basis for care plans because they comprise what individuals with diabetes and related conditions undertake regularly as they self-manage their condition (13). It follows that physical activity, as one of the core contents of diabetes management, is of great significance to diabetes management (14). Physical activity and exercise are often used interchangeably, but these two terms are not synonymous. Physical activity refers to any physical movement produced by the contraction of skeletal muscles, which increases energy expenditure above the basal metabolic rate. It usually refers to activities that can improve health in the classification of physical activity. Exercise, on the other hand, is a specific type of physical activity that is planned, structured, repetitive, and purposefully performed with the aim of improving or maintaining health and physical fitness, which is a subset of physical activity (15). In recent years, a large number of studies at home and abroad have shown that physical activity can help regulate metabolic indexes such as blood sugar, blood fat, blood pressure and body mass through mechanisms such as improving insulin sensitivity, metabolism, vascular function, and reduce the risk of cardiovascular diseases (16, 17). However, the physical activity status of older adults with T2DM is not optimistic. A survey found that 77% of older adults with T2DM in the community were unable to complete the amount of physical activity recommended by the guidelines (18). Most older adults with T2DM live in the community for long-term self-management and community care (19). Community health service institutions, as an essential component of the primary health care system, are important places for implementing standardized management of chronic diseases (20). Therefore, medical staff should pay more attention to older adults with T2DM, who are an important group in community chronic disease management (21).

Influencing the physical activity of older adults with T2DM in the community is a dynamic process involving multi-system interaction and is regulated and coordinated by multiple factors. At present, studies exploring the factors influencing physical activity in people with diabetes mostly adopt single quantitative or qualitative study methods such as qualitative interview, structural equation models, regression analysis, and correlation analysis. For example, Amin et al. (22) found through qualitative interviews that unhealthy emotions would hinder the level of physical activity among older adults with T2DM. Yang et al. (23) discovered through regression analysis that higher education would promote physical activity among older adults with T2DM. However, a single research method is difficult to deeply analyze the complex factors behind them or objectively and accurately reflect the influence degree of each factor. Due to the highly personalized characteristic of physical activity, the mixed-methods study can integrate the advantages of quantitative and qualitative studies, enabling a more comprehensive and accurate understanding of the research subjects. Mixed-methods study refers to a survey model that comprehensively uses qualitative and quantitative studies in a continuous process of exploring the answers to questions, while taking into account philosophy, methodology and practice (24). It links qualitative, quantitative studies and dimensions together, thereby creating a new whole or obtaining a more comprehensive understanding than any single method (25). Therefore, this study adopted a mixed-methods study.

The Multi-theory model (MTM), first introduced by Sharma in 2015 (26), as the fourth-generation theoretical model in the field of health behavior change, not only integrates the essence of the previous three generations of models, but also is characterized by its high accuracy, strong predictive ability and simplicity. The MTM provides a new perspective and effective approach for promoting patients’ behavior change in chronic disease management (27). The MTM divides health behavior changes into two stages-initiation and sustenance, which are, respectively, used to explain the initiation of behavior changes and the sustenance of behavior changes, and simultaneously has strong predictive power (28). The initiation involves three main constructs: participatory dialogue, behavioral confidence, and changes in the physical environment (29). The second component of MTM, the sustenance includes: emotional transformation, practice for change, and changes in the social environment (30). As the fourth-generation theoretical model, the MTM extracts ‘best variables’ from previous theories and forms a unified, concise theoretical framework, which is a good tool for understanding health behavior change (31). Through the results of a meta analysis (32), we found that the MTM is mostly applied in quantitative studies, and only six studies are qualitative studies (33). The MTM is mainly used in healthy behaviors such as smoking cessation, diet, quality of life, vaccine acceptance behavior, and sleep (34–38), as well as patients with gastrointestinal cancer chemotherapy, thyroid cancer, stroke patients and other populations (39–42), meanwhile, it has high predictive ability and practical significance. In addition, at present, there are relatively few studies that explore the factors influencing physical activity in older adults with T2DM based on theory. For the first time, we attempted to apply the MTM to the physical activity of older adults with T2DM in the community. From the perspective of the MTM, we deeply explored the factors influencing physical activity in older adults with T2DM in the community through a mixed-methods study. The text was analyzed by the innovative combination of traditional thematic analysis method and topic modeling (python machine learning) through the qualitative study method, aiming to deeply explore the experiences and views of older adults with T2DM in the community about physical activity. Quantitative study adopted a cross-sectional survey to objectively and efficiently discover causal relationships among data. Finally, the results from the two types of studies were compared to identify differences, similarities, and contradictions. This enabled the mutual verification and supplementation of the research results, compensating for the limitations of a single research method, and ultimately obtain the ultimate results. This study aims to provide practical guidelines for the physical activity management of older adults with T2DM in the community, offer a scientific basis for the government and relevant departments to improve and formulate corresponding policies and gain a new theoretical research perspective for theorists.

## 2 Methods

### 2.1 Study design

**Figure 1:**
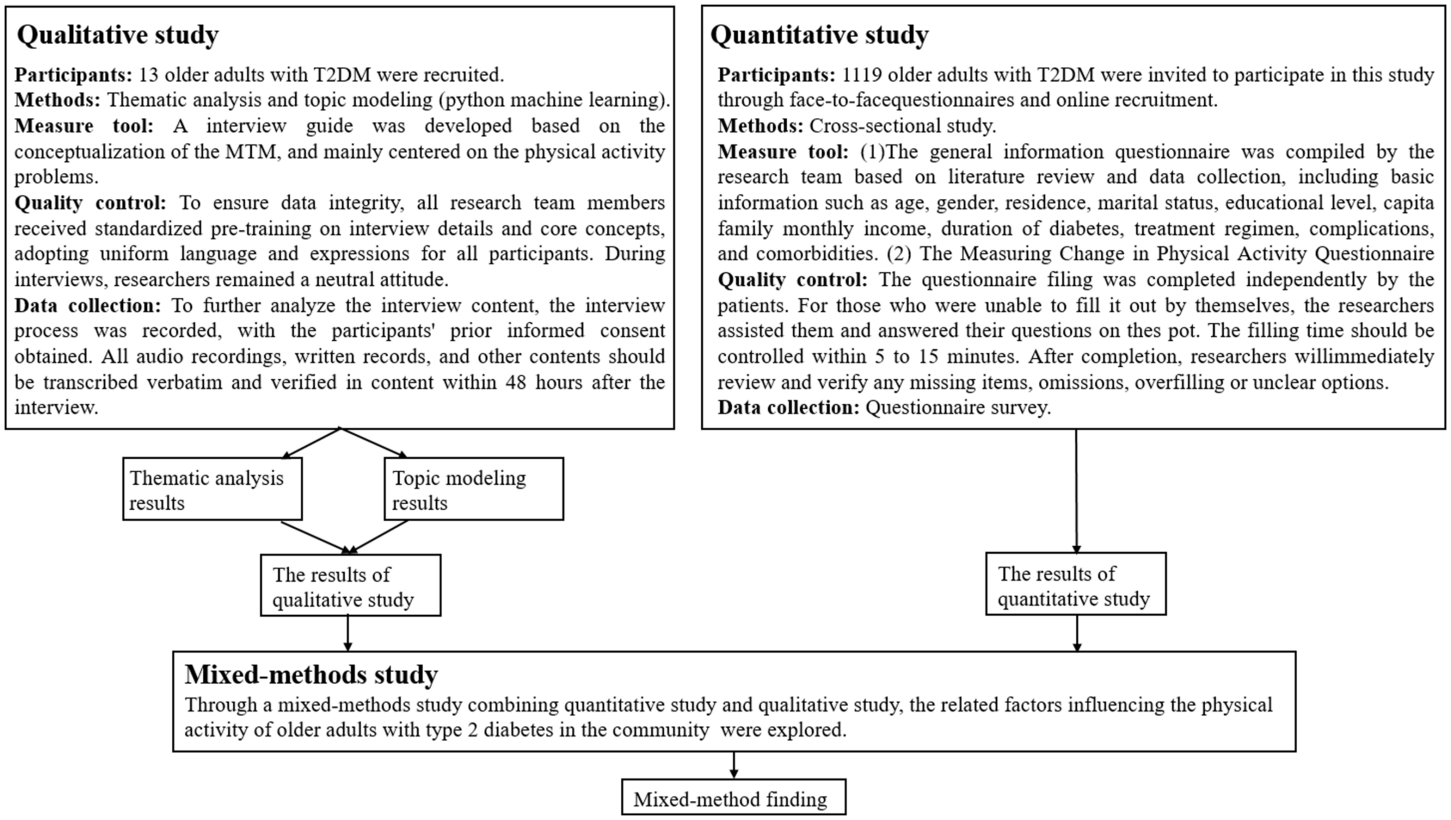
Design of the study using a mixed method of quantitative study and qualitative study.

This research adopts a mixed-methods strategy utilizing a convergent parallel design. Quantitative and qualitative data were collected simultaneously in this design, but they were analysed separately. At the same time, the data were compared and integrated throughout the interpretation phase. Questionnaires and semi-structured face-to-face interviews were used to collect data, respectively. In the qualitative component, an interview guide was developed based on the MTM to investigate the influence mechanism of physical activity in older adults with T2DM. The quantitative part consisted of a cross-sectional survey incorporating demographic variables to examine the influence mechanism. Through the use of a convergent parallel design, the study sought to achieve a comprehensive understand of the research problem by integrating qualitative and quantitative findings (Figure 1).

### 2.2 Step 1: descriptive qualitative study

#### 2.2.1 Materials and methods

The Standards for Reporting Qualitative Research (SRQR) guidelines was used as the reporting guideline (43). The interview guide was developed based on the conceptualization of the MTM, and experts were invited to agree on the details of the interview guide after discussion. Table 1 is the latest guidelines for the interviews with the participants. Semi-structured interviews were applied in this study to ensure that participants could provide detailed explanations of the influences of physical activity on diabetes. In this study, two methods were employed to analyze the interview data, such as thematic analysis and topic modeling (python machine learning), in order to gain a more comprehensive and detailed understanding of the interview results (44, 45). We conducted a comparative analysis of the results of these two methods, thereby identifying the final factors related to the physical activity of older adults with T2DM in the community.

**Table 1:** Guidelines for the interviews with the participants.

Stage	Constructs	Content
Initiation	Participatory dialogue	Could you discuss the advantages of physical activity? Could you discuss the disadvantages of physical activity?
	Behavioural confidence	Do you think you could successfully initiate and sustain physical activity change? What could cause you to become aware of this behavior?
	Changes in the physical environment	What physical environments are there in your immediate surroundings that might influence a change in your physical activity? What sort of effect does it produce? What changes would you like to see made to support you in initiating and sustaining the change for physical activity?
Sustenance	Emotional transformation	What kinds of emotional shifts or mood swings did you encounter when your physical activity changed during the sustenance? How did you get over your bad feelings? (This can be explained as starting with sustenance and ending with it.)
	Practice for change	Do you actively consider these actions when it comes to physical activity change practices? (For instance, how do you define right and good? (For instance, how do you define right and wrong, as well as good and bad?) If you give it some thought, will your next practice change as a result?).
	Changes in the social environment	What kind of social support did you receive during the sustenance of your physical activity change? What specific assistance did these supports provide you?

##### 2.2.1.1 The inclusion and exclusion criteria

This qualitative study was conducted in March 2024. In order to promote the progress of the research, the researchers randomly selected a community from the school community practice site. Purposive sampling was used. The community leader posted the recruitment information in the wechat groups for diabetes patients in the community. Participants were screened according to the inclusion and exclusion criteria. The inclusion criteria for participants were as follows: (1) a diagnosis of T2DM, in accordance with *the Guideline for the Management of Diabetes Mellitus in the* Elderly *in China (2024 edition)* (46); (2) aged 60 years or older (47); and (3) having clear consciousness without any intellectual impairments. Meanwhile, the exclusion criteria included: (1) combined with other severe comorbidities, such as malignant tumors; (2) facing language communication challenges; (3) reluctance to take part in the study; and (4) impaired consciousness.

##### 2.2.1.2 Quality control

To ensure data integrity, all research team members received standardized pre-training on interview details and core concepts, adopting uniform language and expressions for all participants(Huancheng Su and Jinli Guo are the trainers for the pre-training, and all the other members of the team (Panpan Huai, Bo Zhang, Jingjing Sun, Rui Xu, Linghui Zhang, Xiao Qiao, Weili Sun, and Hui Yang) participate in the pre-training). Before the interview, researchers verified the self-reported information of participants according to the chronic disease records in the community health service center to ensure that the information is accurate. Eligible individuals provided their contact details. Interview arrangements were coordinated in advance, and one-on-one interviews were conducted in private spaces, such as the community health education room or unoccupied chronic disease management wards. During interviews, researchers remained a neutral attitude. For participants who were unable to complete the questionnaire independently, researchers read out questions without offering any hints, following standardized instructions. Check on the spot whether the patients have filled out the questionnaire completely. If there are any omissions, supplement them in time to ensure the authenticity and reliability of the questionnaire data information of the research subjects. After the interview, two researchers independently transcribed, analyzed, and coded the data. Discrepancies were resolved through group discussions (all members of the team participate in the group discussion). Additionally, participants also reviewed the finalized transcripts for accuracy. When collecting questionnaires, any missing items were promptly followed up with participants to maintain data quality.

##### 2.2.1.3 Ethical consideration

Our study was approved in advance by the Ethics Committee of Balingqiao Community Health Service Center, Xinghualing District, Taiyuan City (approval number:20230001). This research adheres to the principle of informed consent. Participants voluntarily participated in the study. Before the study began, the researchers provided a detailed introduction and explanation of the background, purpose, content, potential risks and benefits of the study to the participants, and they signed a written informed consent form. All data were kept confidential. Throughout the entire study process, participants had the right to raise questions at any time and withdraw from the study.

#### 2.2.2 Data collection

The researchers conducted one-on-one and face-to-face interviews with the participants. Each interview lasted approximately half an hour. Before each interview, each participant signed a written informed consent form. To further analyze the interview content, the interview process was recorded, with the participants’ prior informed consent obtained. All audio recordings, written records, and other contents should be transcribed verbatim and verified in content within 48 h after the interview. When we reached consensus on the data saturation of the framework themes in the sample, the data collection work was completed and the sample size was determined (48). After the interviews, the completeness and accuracy of the interview content were determined through group discussions.

#### 2.2.3 Data analysis

##### 2.2.3.1 Thematic analysis

A common usual qualitative methodology is thematic analysis (49). In order to better identify the focus of the research issue, we first conducted a word frequency search. Based on the transcripts, we used subject matter analysis to manually classify the interview data. The process involved (1) familiarizing with the data, (2) developing an initial set of codes, (3) exploring themes, (4) reviewing themes, (5) defining themes, and (6) generating the report (49). Names were substituted with numbers to portray the results in an anonymous manner. The participants’ nonverbal cues were noted at the appropriate positions, and the data were imported into the NVivo 14.0 software for organization and analysis. Two researchers conducted the analysis separately to guarantee interpretive validity and transparency. The transcribed interviews were analyzed using the MTM framework as a guide. Then, two researchers argued about the first code development as well as the subsequent definition and creation of the subject matter until they came to a unified decision to improve the analysis’s rigor (50). We selected specific examples from participant responses to highlight each theme in order to make it easier to understand their significance.

However, those qualitative analyses also seem to depend on people’s perceptions (51). Additionally, researchers could use a significant amount of cognitive resources reading and evaluating transcripts continually, participating in rounds of discussion, and producing extensive transcripts. We supplemented the thematic analysis using topic modeling to provide fresh perspectives because the scope of the interviews in this study may have increased the likelihood of omissions.

##### 2.2.3.2 Topic modeling

Topic modeling is also one of the methods of text analysis (37, 52). Topic modeling may yield a number of word clusters that are the fundamental ideas for determining the fundamental and underlying elements of linguistic data (53). Topic modeling has been used in a number of fields, most notably social media analysis (54) and couples therapy research (53). Topic modeling was used in this work as a supplement to interview transcript analysis (33).

Multiple word clusters which serve as the key concepts for identifying the essential and underlying components of linguistic data may be obtained from topic modeling (52). Several disciplines, notably social media analysis (37) and couples therapy research (52), have utilized topic modeling. In the present study, topic modeling was implemented as an adjunct to the transcript analysis of interviews. Using the combined use of thematic analysis and topic modeling, we are capable of offering exciting and comprehensive viewpoints that reinforce research questions (31).

For the purpose of topic modeling, this study gathered every participant’s word that was recorded during the interviews. To ensure the accuracy and readability of the analytical results, all texts were first cleaned up using python. Word segmentation, word elimination (i.e., stop words and words shorter than two characters were deleted), and special symbol deletion were among the preprocessing techniques used. By doing this, the volume of irrelevant phrases and noise may be reduced, potentially improving the performance of the topic model algorithm (55). This led to the acquisition of the final dataset for topic modeling. The latent Dirichlet allocation (LDA) algorithm was then used to examine the dataset’s main themes and structures. A common topic modeling method called LDA can be used to examine several subjects within a collection of texts. The probabilistic LDA approach makes it easier to infer latent theme structures from documents that do not have manual labels or previous information (54–56). The Mallet version of LDA, which is believed to perform better than LDA, is implemented by the python package Gensim (57). Therefore, we used the Mallet version of LDA to build the topic model and search for thematic patterns in our data.

The number of themes is one important aspect influencing the model’s performance. To establish the optimal number, we explored with two to thirty themes, calculating the perplexity for each model. Perplexity serves as a gauge for the LDA topic model’s degree of fit and quantifies the model’s prediction power over the data (58). A lower perplexity indicates a more accurate model. Based on the confusion, the ideal number of subjects was chosen after the final theme model was validated. PyLDAvis was then used to view the topic model output. A graphical user interface for conceptualizing inter-topic distance is provided by the python application PyLDAvis (59).

The final step of topic modeling was analyzing, identifying, and characterizing subjects based on the results of the LDA algorithm. Two authors discussed the top 30 keywords for each subject and reviewed the phrases that matched each topic in the interview transcripts after the coherence score recommended subjects. After that, they agreed on the title and synopsis for each topic.

### 2.3 Step 2: quantitative study

#### 2.3.1 Participants

The quantitative study protocol adhered to the STROBE statement (60), which was conducted from March 2024 to June 2024. The participants were older adults with T2DM living in the community of Shanxi Province, China (including participants selected in the qualitative study). To reduce bias, a unified and standardized description was adopted for older adults with T2DM in the community to introduce the purpose and content of the study, ensuring that older adults with T2DM in the community had a full understanding of the study. Furthermore, the process of collecting questionnaires strictly selected the research participants in accordance with the inclusion and exclusion criteria. The inclusion and exclusion criteria were consistent with those of the qualitative study. In the research exploring the factors of relevant variables, the sample size should be at least 5 to 10 times the number of variables (61). The questionnaire of this study contains 11 items. Considering that 20% of the samples are inefficient, the calculation requires a sample size of 66 to 132 cases. Ultimately, 1,119 older adults with T2DM were invited to participate in this study through face-to-face questionnaires and online recruitment. The questionnaire filling was completed independently by the patients. For those who were unable to fill it out by themselves, the researchers assisted them and answered their questions on the spot. The filling time should be controlled within 5 to 15 min. After completion, researchers will immediately review and verify any missing items, omissions, overfilling or unclear options. This study was approved by the Ethics Committee of the Second Hospital of Shanxi Medical University (approval number:2023YX288). All of the participants or their legal guardians gave their informed consent to participate.

#### 2.3.2 Measures

##### 2.3.2.1 The general information questionnaire

The general information questionnaire was compiled by the research team based on literature review and data collection, including basic information such as age, gender, residence, marital status, educational level, capita family monthly income, duration of diabetes, treatment regimen, complications, and comorbidities.

##### 2.3.2.2 Measuring change in physical activity questionnaire

Intentions to engage in physical activity were evaluated using the Measuring Change in Physical Activity Questionnaire (MCPAQ). It was originally developed in English based on the MTM construct by Sharma (62). The higher the scores of changes in physical activity, the greater the likelihood of conducting physical activity behavior change. Yang et al. (63) obtained authorization from the original authors of the MCPAQ and conducted a cross-cultural adaptation to develop a Chinese version of the scale. This version was validated in hypertensive patients and demonstrated good reliability and validity: Cronbach’s alpha was 0.911 for the overall scale. The scale is considered broadly applicable across diverse populations.

#### 2.3.3 Statistical analyses

Excel 2016 was used for data entry, while SPSS 25.0 statistical software was used for data analysis. The data was tested for normalcy using Shapiro–Wilk. In terms of statistics, the mean and standard deviation (*SD*) were used to characterize the normal quantitative data. The median and interquartile range (*IQR*) applied to the quantitative data that had an irregular distribution. In terms of statistics, the qualitative data was expressed as frequency (*n*) and percentage (%). The two independent samples *t*-test or one-way analysis of variance is used for comparison of measurement data that meet the requirements for homogeneity of variance and normal distribution. Using multiple linear regression analysis, the contributing factors were examined. Statistical significance was defined as a *p* value of less than 0.05.

## 3 Results

### 3.1 The results of qualitative study

In this study, we carried out 13 interviews. Table 2 shows the demographic information of our interview participants.

**Table 2:** Demographic information of interview participants.

Sociodemographic characteristics	*N* = 13
Age ( $\bar{X}$ ± SD)	71.85 ± 4.98
Gender *n* (%)	
Male	8 (61.54%)
Female	5 (38.46%)
Educational level *n* (%)	
Elementary school and below	0
Middle school	8 (61.54%)
High school or technical secondary school	4 (30.77%)
College or bachelor degree	1 (7.69%)
Master and above	0
Residence *n* (%)	
Urban	12 (92.31%)
Rural	1 (7.69%)
Capita family monthly income (yuan) *n* (%)	
<1,000	1 (7.69%)
1,000–3,000	5 (38.46%)
>3,000	7 (53.85%)
Living status *n* (%)	
Living alone	2 (15.38%)
Living with others	11 (84.62%)
Marital status *n* (%)	
Single	0
Married	11 (84.62)
Divorced	1 (7.69%)
Widowed	1 (7.69%)
Treatment regimen	
None	3 (23.08%)
Oral hypoglycemic agent	8 (61.54%)
Insulin	0
Oral hypoglycemic agent + insulin	2 (15.38%)
Others	0
Duration of diabetes (years) (X̄ ± SD)	10.2 ± 9.3
Comorbidity n (%)	
None	5 (38.46%)
Hypertension	7 (53.85%)
Dyslipidemia	1 (7.69%)
Coronary heart disease	1 (7.69%)
Stroke	0
Complication *n* (%)	
None	7 (53.85%)
Cardiovascular and cerebrovascular diseases	1 (7.69%)
Diabetic nephropathy	0
Diabetic ophthalmopathy	4 (30.77%)
Diabetic foot	0
Diabetic neuropathy	0
Diabetic dermopathy	1 (7.69%)

#### 3.1.1 Thematic analysis results

**Figure 2:**
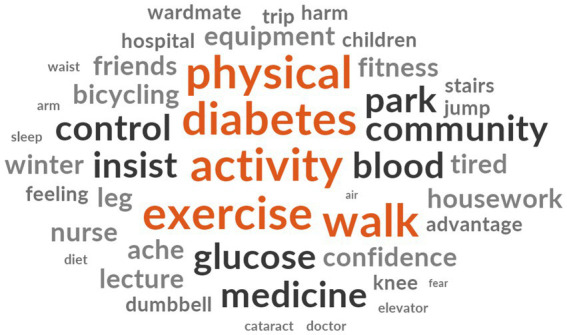
Word frequency diagram.

After the interview, a thematic analysis was carried out utilizing the MTM as the theoretical framework to examine the interview transcripts. Figure 2 is word frequency diagram. According to this, the interview mainly focused on physical activity (*n* = 98), diabetes (*n* = 66), walk (*n* = 59), exercise (*n* = 50), and medicine (*n* = 47). After generating thirty-one initial codes, we found seventeen important factors (Table 3).

**Table 3:** The results of thematic analysis.

Theroy	Stage	The constructs	The key factors	Examples
MTM	Initiation (three primary constructs)	Participatory dialogue	*Benefits of moderate physical activity* (physical health, mental health) *The effects of Intense physical activity/excessive physical activity intensity*	I will feel some sweating, refreshed, my mind becomes more agile, I find food more delicious, my sleep improves, I fall asleep faster and it’s easier now, and my sleep time is longer. I think physical activity is a good thing. It is beneficial to physical and mental health. (Interview #9) However, in the process of activity, muscle strain, ligament strain, and accidental fall may occur. If today I have a large amount of activity, I will feel very tired and tired. (Interview #9)
		Behavioral confidence	*Internal self-confidence*	There is no harm in doing physical activities. They are all benefits, and I am quite confident that I will keep doing them. (Interview #6)
		Changes in the physical environment	*Convenience and accessibility of fitness facilities* *Accessibility of health equipment* (e.g., glucose meters) *Accessibility and convenience of network health information resources*	There’re fitness facilities in the neighborhood. No, I do not want to use it. Sometimes used to turn the arms and shoulders, the fitness facilities are not suitable for us old people, are made for young people, honestly, old people are afraid of falling, you know? You said a fall would send you straight to the crematorium. (Interview #6) If my blood sugar level is abnormal during this period, I would feel abnormal myself. Or if I change my diet today and the food I eat is different from usual, I would measure my blood sugar. There is a blood glucose meter at home. (Interview #7) I will pay attention to the live-streaming videos of traditional Chinese medicine on Douyin, and learn about the acupoint massage techniques of traditional Chinese medicine from the live-streaming sessions to promote my physical health. (Interview #2)
	Sustenance (three primary constructs)	Emotional transformation	*Shifts in disease acceptance*	I usually have no mood changes, the mood is very stable, and no one quarrel with me, nor make me angry, very plain. Is just the beginning of diabetes when the mood is not good, thinking that others are OK, I have diabetes, unhappy, and then the blood sugar stabilized, a long time I also put down, do not want to, anyway, it is every day to take medicine, there is nothing. (Interview #2)
		Practice for change	*The comprehensiveness of physical activity behavior types* *The persistence of physical activity*	I usually walk, but recently I started playing with dumbbells. (Interview #8) When it was just drizzling, I went out with an umbrella. But when it got heavier, I did not go out. At home, I just shook my neck and back, shook my head and twisted my waist, doing something like a health exercise. (Interview #4)
		Changes in the social environment	*Social support from peers and family members* *Being able to establish helpful social relationships*	My family members are all quite supportive of my doing sports. However, they will not accompany me to do sports. I always go cycling alone. But they know that my legs are not in good condition, so they buy me calcium tablets, amino acids and other things for me to take every day. (Interview #10) The doctors and nurses in the community are really great. They regularly monitor our blood sugar levels for us, tell us how to eat and how to physical activity, and also give us lectures from time to time. (Interview #10)
Beyond MTM		Cultures	*Educational level* *Traditional conception* *Family atmosphere*	There are usually diabetes lectures. Sometimes I can understand them. But mainly, when encountering those technical terms, I just cannot follow along. (Interview #3) No one can be better than oneself. Take good care of one’s own health and do not burden one’s children. That’s exactly what I think now. Do not cause trouble for the children. Do not lose memory or lose the ability to act independently when you are 90. Just strive to be able to take care of oneself and remain independent until the age of 100. Anyway, now it’s enough to manage oneself well and take good care of one’s own health. Right? I really mean that I do not want to burden my children. You all just go to work well, do a good job, and raise your descendants well. (Interview #6) For me, diabetes, I know all too well its harmful effects because several of my family members have died due to diabetes. They all had diabetes (spasmodic sobs). There have been too many deaths of my relatives due to this disease. You know, there are all kinds of symptoms associated with it. I’ve seen it all, so I know the harmful effects of diabetes very well. That’s why I pay special attention to diabetes. (Interview #7)
		Stimulation	*Stimulation*	I dare not move. I’m afraid of falling down. I’ve fallen down several times before. People said that if I fell down one more time, I would never be able to stand up. I have a cerebral infarction. It was discovered after I broke my bones when I fell down once. Since then, I have been even more afraid of going out in winter. My daughter calls me, but I dare not come out. I’m afraid that if I fall down again, what should I do? (Interview #13) Previously, I was not very active. Since I got diabetes, I started to be active because I was afraid of having high blood sugar levels. (Interview #1)
		Intrinsic disease	*Comorbidity* (e.g., myocardial infarction) *Common health problems in older adults* (e.g., backache, arthromeningitis)	In the past, I used to do boxing and such. But now that I have heart disease, I do not do those strenuous exercises anymore. Because when you do them, your breath just will not be enough. You can only walk. But you can only walk at a certain speed. When you start to breathe heavily, you’ll slow down. (Interview #5) My lower back pain has prevented me from doing some other activities. Moreover, I do not do any special exercise deliberately to treat my diabetes. The main thing is just to take walks. (Interview #9)

#### 3.1.2 Topic modeling results

**Figure 3:**
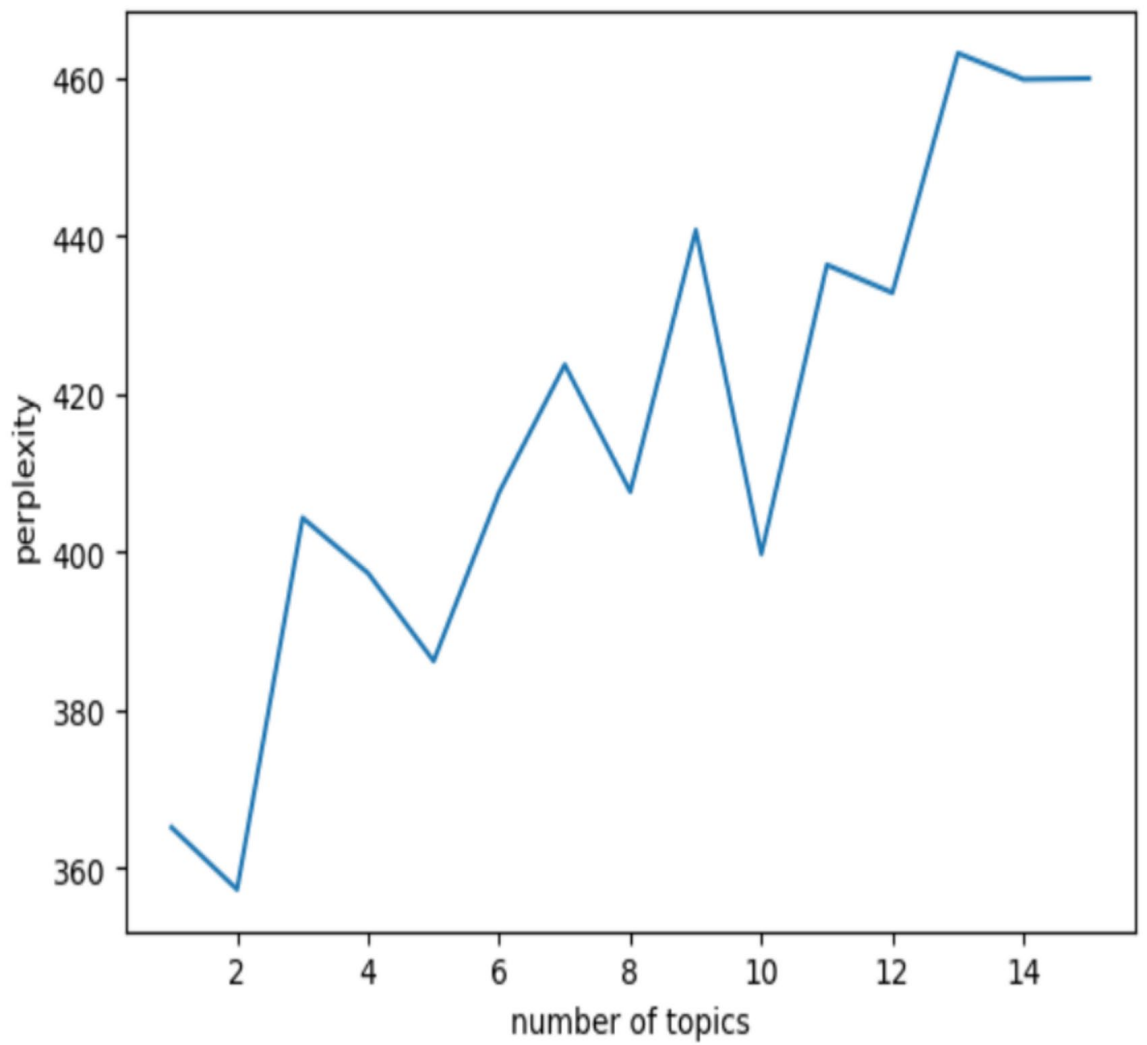
Perplexity scores for different number of topics.

Qualitative study determined the perplexity score for each topic model to determine the ideal number of topics for topic modeling. The perplexity scores of each topic model created using various topics, are shown in Figure 3. Firstly, when the number of topics is set to 8, it can be seen from the low perplexity score that the topic model performs efficiently. Secondly, due to the small scale of our corpus, too many topics may mask the main viewpoints of the text itself, while too few topics may make it difficult to extract the main viewpoints. Therefore, the 8 topics have the most significant topics while avoiding the risk of confusing the main focus of the research, which contrasts with the number of other topics with lower perplexity score (such as 5 and 10).

**Figure 4:**
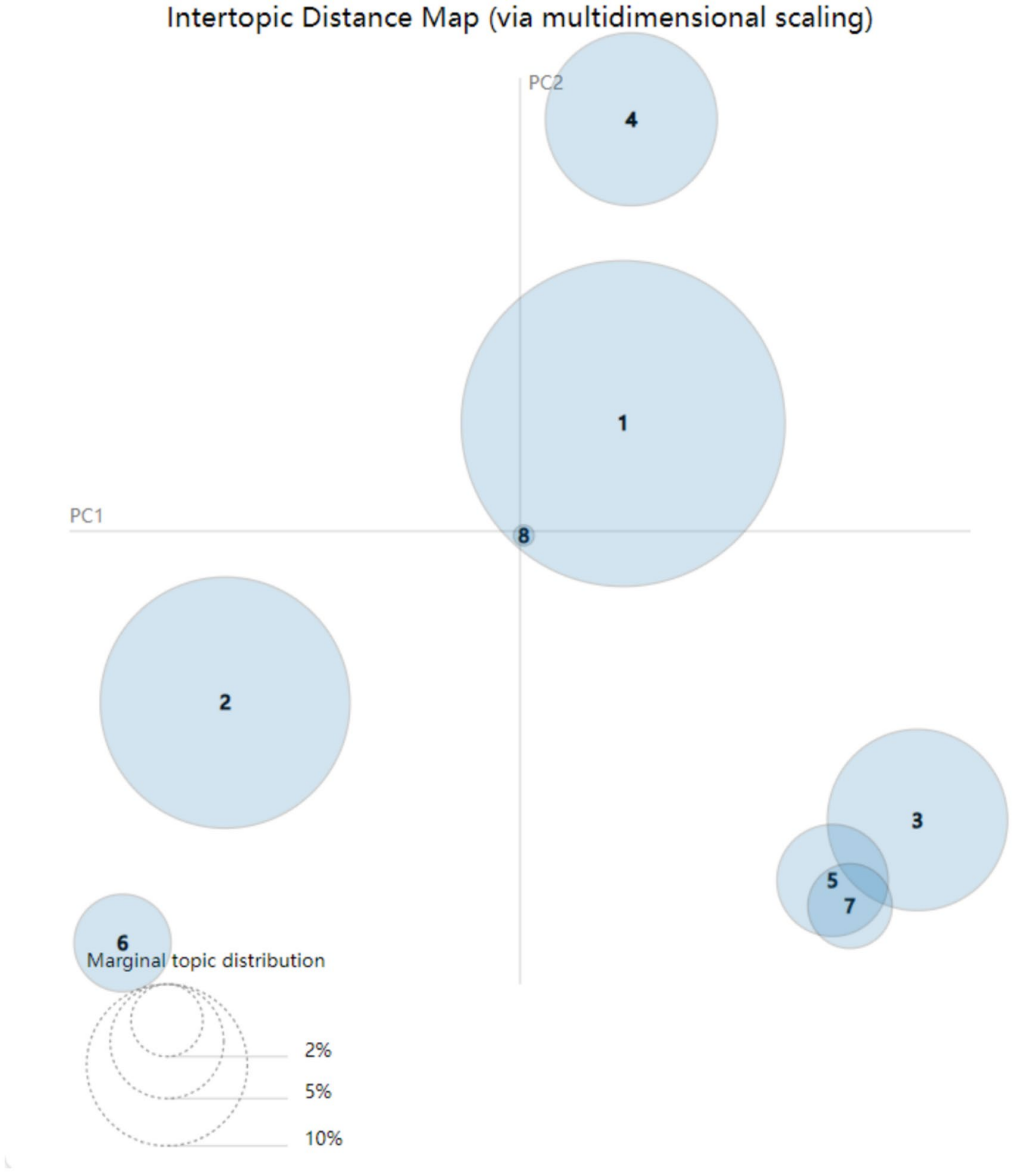
The inter-topic distance map.

Figure 4 is the inter-topic distance map. In the inter-topic distance map, each bubble represents a topic, and the coverage of the topic is reflected by the area of the bubbles. The relatively large and non-overlapping bubbles in the figure indicate the appropriate topic modeling results. From Topic 1 to Topic 8, each bubble represents a unique topic. The bubbles in Figure 4 are relatively large and most of them do not overlap. Therefore, based on the perplexity score and the inter-topic distance map, it is ultimately determined that eight topics are the ideal number for topic modeling.

Table 4 presents eight of the top 30 topic words along with their related terms, and it is also the result of topic modeling. Analysis shows that the number of topics is the same as the number indicated in the inter-topic distance map. After the research team conducted grouping, review and discussion on these eight topics through topic modeling, we determined these eight topics as the final category of topics, and assigned labels to each topic, as shown in Table 5.

**Table 4:** The topic model result with eight topics and the relevant words.

Topic	The top 30 relevant words
1	Bicycling; instrument; mood; older adults; mobile phone; buy vegetables; activity; disease; lumbar vertebra; terminal; factor; weather; method; kind; concern; spouse; housework; walk; clean the floor; age; caution; go for a walk; ache; season; place; running; corridor; dumbbell; elevator; beneficial
2	Adjustment; doctor; adjust; patient; spouse; blood pressure; in hospital; ear; diet; harm; kidney; glucose; hypoglycemia; sustained release tablet; urine protein; cube sugar; nephropathy; egg; porridge; avoid certain food; rule; breakfast; brother; family; age; housework; shoulder; milk; physician; beneficial
3	Doctor; diet; buy vegetables; sing; daughter; hospital; son; inspection; do; older adults; digestion; arthromeningitis; arm; breakfast; limosis; elevator; steamed stuffed bun; children; blood fat; sicken; nurse; glucometer; apple; aged; kid; eye; lecture; mood; emotion; porridge
4	Myocardial infarction; Hospitalization; mobile phone; amount of exercise; eight trigrams boxing; heart; breakfast; age; problem; doctor; nurse; education background; air; playing Mahjong; heart disease; factor; Tai Chi; regulate blood sugar level; way; leg pressing; circulation; speed; strenuous exercise; reduce blood glucose; skin; belief; friend; fall ill; thought; patient
5	Epidemic; wardmate; knee; family; emotion; education; hyperglycemia; neck; Hospitalization; daughter; diabetic; belief; bus; disease; rainy day; heart; spouse; thought; operation; WeChat; outdoor exercise; mood; patient; attitude; myocardial infarction; psychologist; cardiopulmonary; sicken; heart disease; strength
6	Walk; weather; housework; lung; inflammation; emotion; video; friend; immunity; anger; at leisure; voice; fall ill; advantage; acupoint; mood; Lecture; attitude; cardiopulmonary; outdoor exercise; myocardial infarction; psychologist; patient; sicken; avoid certain food; heart; thought; heart disease; strength; WeChat
7	Egg; brother; WeChat; mood; myocardial infarction; psychologist; cardiopulmonary; heart; cardiopathy; avoid certain food; manner; thought; sicken; patient; feeling; outdoor exercise; operation; mobile phone; arm; poker; playing Mahjong; strength; square; nurse; young people; children; terminal; ration; objective condition; family
8	Spouse; square; eye; Hospitalization; children; old man; coarse food grain; cataract; eight trigrams boxing; daughter; diet; friend; couple; gastric carcinoma; buy medicine; operation; hospital; family; steamed bread; have an operation; chemotherapy; fluctuation; effective; equipment; environment; inheritance; appetite; limosis; issue; nurse

**Table 5:** Eight topics produced by LDA with labeled names and examples.

Topics	Examples
Topic #1 *Fitness facilities in a physical environment And Other forms of physical activity other than the use of equipment*	I hope there could be some specialized exercise equipment, specifically the kind that is designed for people like us who have poor lumbar spine conditions. Because I think most of the fitness equipment and activity facilities in the park are in bad condition, and some of them I just cannot use at all. For instance, some of those arm-strengthening equipment were too high for me to reach and I accidentally fell down when trying to reach them. Also, there are too many people on some fitness equipment, like they are sitting there with their phones and playing games instead of exercising. They just occupy the seats and sit there. (Interview #9) I like to play with dumbbells. At the beginning, for three or 4 months, I practiced every day, doing 90 reps each time and three times a day. (Interview #8) Our community used to have fitness equipment. But later, it was supposed to be renovated in 2 or 3 years. However, the renovation wasn’t completed because the community is too large. These days, the community is being improved. Since it was demolished, the lower part of the community now has over ten buildings. They have just finished repairing the ground of the buildings and have not installed the equipment yet. So now, I can only walk around. (Interview #3)
Topic #2 *Fear of complications caused by physical activity*	I’m always afraid of hypoglycemia when I go out for physical activity. I feel my heart racing, my hands shaking and sweating, especially sweating. I sweat so much that my clothes get soaked. So I always carry glucose cubes in my pocket when I go out, just in case. If that’s not available, cola will do too. Cola can also raise blood sugar very quickly. (Interview #7) We older adults cannot do vigorous activities. We should do things in moderation. Otherwise, we are likely to have back pain and be afraid of getting kidney diseases. (Interview #9)
Topic #3 *Physical activity is limited by an intrinsic disease*	Besides diabetes, I also have synovitis, which cannot be cured. I can only pay attention to keeping warm. So when it gets cold in winter, I do less exercise outside. (Interview #3) Because I have cataracts and my vision is blurry, my legs also hurt. I have not gone out for a whole winter, fearing I might fall. I do not move around much at home either. (Interview #13)
Topic #4 *Focus on physical activity itself*	I used to enjoy boxing, but now I have heart disease and cannot do vigorous activities. Otherwise, I’ll be out of breath. As people get older, they tend to have some minor health issues. We cannot be as active as young people. We have to be moderate in our exercise. We cannot overdo it, or we’ll risk muscle injuries, ligament strains and exhaustion. (Interview #5) The intensity of my activities is relatively low. My main activities include: buying groceries, taking walks in the park, climbing six floors of stairs to go upstairs, cleaning, and doing some housework. I do not cook. They are all fragmented activities. As for leisure activities, I go on trips and climb mountains every year. I have been doing this for several years. (Interview #9) I did not use the fitness equipment in the community because I found it troublesome to use and there were not many types, which did not quite meet the needs of older adults. I prefer simple exercise methods like walking or cycling. (Interview #10) I used to insist on going outside every morning to do square dancing and practice Baduanjin, but now I cannot do that anymore. My old man at home is blind, so I have to stay at home and take care of him. Sometimes I push him out to enjoy the sun and take a walk in the community. I do not go out to exercise by myself. (Interview #12) I enjoy reading and can sit for 2 h straight. The best way for me is to use an acupressure mat. I stand on it and move my feet back and forth. It boosts blood circulation and lowers blood sugar very quickly. I do not do much other exercise because if it’s too intense, I cannot catch my breath. (Interview #5)
Topic #5 *Uncontrollable factors that affect physical activity* (e.g., weather, air quality, disease prevalence, interpersonal interactions, etc.)	Before the pandemic, I used to go to the fitness equipment area in my community. But after the outbreak, I stopped going. Later, I got used to it and did not go there anymore. Instead, I just walk more. (Interview #4) At first, I would go to Longtan Park, stretch my hands and take big strides with my legs. But when the epidemic started, I stopped going. Now there are also some places in the community, but there are too many people, all old men and women, so I do not go there to join in the fun anymore. When I walk, if it’s far, I take the bus. (Interview #4) When it was just drizzling, I went out with an umbrella. But when it got heavier, I did not go out. At home, I just shook my neck and back, shook my head and twisted my waist, doing something like a health exercise. (Interview #4) Almost every day, as long as it does not rain or blow, this kind of weather is just perfect for going out and doing activities. (Interview #8)
Topic #6 *Perceive the benefits and disadvantages of physical activity*	Physical activity has certain benefits for maintaining stable blood sugar levels. Besides, it can also help pass the time and enhance the body’s immunity. (Interview #2) I’m not sure whether physical activity can lower blood sugar, but I do enjoy activities and feel better after them, and my mental state will also be better. Moreover, my physical strength and endurance are not inferior to those of my peers, and even seem younger than them. Physical activity makes me feel energetic and much younger. (Interview #11) The benefits of physical activity far outweigh the drawbacks. During the activity, there might be muscle strain, ligament sprain, and accidental falls. If I say that today my activity intensity was high, I would feel very tired and exhausted. (Interview #9)
Topic #7 *Emotion regulation types*	When I’m in a bad mood, I play mahjong. Usually, I play mahjong for about 4 h. Sometimes I play with my phone, chatting with people on WeChat, watching short videos, and playing for two or 3 h. Sometimes I even sit up on my side. I like reading books. Even when sitting, I can sit for 2 h. (Interview #5)
Topic #8 *Social support for physical activity*	The family members did pay attention to my blood sugar. My daughter is in the pharmaceutical business. She would ask me if there was any medicine left. If there wasn’t, she would buy it for me. At first, it was the fellow patients who told me to get moving. The fellow patients organized activities together. So I went. Actually, there wasn’t much wrong with it. It was like getting a health check-up once. It was just that I had company during my hospital stay. (Interview #4) I often come to the community hospital. The doctors here know my condition very well. I often come to see them regularly. They always tell me a lot of knowledge about diseases. This is very helpful to me. (Interview #9) On the mobile phone, there are some information about diabetes and exercise. When I was hospitalized, I happened to catch the Diabetes Day. The doctors and nurses who introduced it were very clear. Exercises like Baduanjin and Tai Chi, I have been practicing these since childhood. So for me, they are not unfamiliar. I also know which method can lower blood sugar faster. I only do a few movements of Baduanjin and Tai Chi. (Interview #5)

#### 3.1.3 Comparison of thematic analysis and topic modeling results

In the emotional transformation, the results of thematic analysis mainly focus on the influence of the changes in patients’ emotions at the initial stage of the disease on the changes in physical activity. However, topic modeling not only pays attention to the emotional transformation at the beginning of the disease, the emotional changes of the whole course of T2DM and even the emotional changes caused by other factors also focuses on how patients regulate their emotions to promote the initiation and sustenance of changes in physical activity. In Beyond MTM, the topic modeling also adds *Uncontrollable factors that affect physical activity*, emphasizing that uncontrollable factors such as weather, air quality (for example: haze), prevalence of disease (for example: SARS) and interpersonal communication can also affect the change in physical activity; The topic modeling also supplements the *fear of complications caused by physical activity*. It was emphasized that older adults are afraid that physical activities may cause diabetic complications such as foot injuries or even lead to infections after foot ulcers, thereby affecting their physical activity. In the behavioral confidence, the thematic analysis mentioned the *Internal self-confidence*, while the topic modeling did not analyze this topic. In Beyond MTM, the thematic analysis mentioned the influence of *Stimulation* and *Cultures* on physical activity, while topic modeling did not analyze these two topics. Topic modeling and thematic analysis complement each other to form an efficient and systematic process of text processing and analysis. The close combination of the two in the field of text mining and information parsing not only improves the efficiency and accuracy of data processing, but also broadens the depth and breadth of text content parsing, providing strong technical support and decision-making basis for scientific research and other fields. Combining the results of thematic analysis and topic modeling, we analyzed the similarities and differences between them and identified 11 topics including 21 factors as the set of qualitative study results (Table 6).

**Table 6:** The key influencing factors derived from two different analysis methods.

Theroy	Stage	Constructs	Qualitative research	
			Thematic analysis	Topic modeling
MTM	Initiation (three primary constructs)	(1) Participatory dialogue	*Benefits of moderate physical activity* (physical health, mental health) *The effects of Intense physical activity/ excessive physical activity intensity*	*Focus on physical activity itself* *Perceive the benefits and disadvantages of physical activity*
		(2) Behavioral confidence	*Internal self-confidence*	Not found
		(3) Changes in the physical environment	*Convenience and accessibility of fitness facilities* *Accessibility of health equipment* (e.g., glucose meters) *Accessibility and convenience of network health information resources*	*Fitness facilities in a physical environment*
	Sustenance (three primary constructs)	(1) Emotional transformation	*Shifts in disease acceptance*	*Emotion regulation types*
		(2) Practice for change	*The comprehensiveness of physical activity behavior types* *The persistence of physical activity*	*Other forms of physical activity other than the use of equipment*
		(3) Changes in the social environment	*Social support from peers and family members*	*Social support for physical activity*
			*Being able to establish helpful social relationships*	
Beyond MTM	Intrinsic disease		*Comorbidity* *Common health problems in older adults*	*Physical activity is limited by an intrinsic disease*
	Stimulation		*Stimulation*	Not found
	Cultures		*Educational level*	Not found
			*Traditional conception*	
			*Family atmosphere*	
	Uncontrollable factors		Not found	*Uncontrollable factors that affect physical activity* (e.g.*, weather, air quality, disease prevalence, interpersonal interactions, etc.*)
	Complication		Not found	*Fear of complications caused by physical activity*

### 3.2 Quantitative study results

#### 3.2.1 The general information of participants and the scores of changes in physical activity

In this quantitative study, a total of 1,119 patients were included. The results of the univariate analysis indicated that there were statistically significant differences the scores change in physical activity among older adults with T2DM in terms of age, residence, educational level, living status, capita family monthly income, duration of diabetes, complication, comorbidity, as well as treatment regimens (*p* < 0.05) (Table 7).

**Table 7:** General data of older adults with type 2 diabetes and univariate analysis of change in physical activity scores (*N* = 1,119).

Sociodemographic characteristics	*n* (%)	The score of change in physical activity ( $\bar{X}$ ± SD)	Test statistic	*P* value
Gender				
Male	537	88.02±30.59	*t* = 0.059	0.953
Female	582	87.91 ± 28.79		
Age (years)				
≥60, <70	747	87.97 ± 29.64	*F* = 6.304	0.002
≥70, <80	282	84.88 ± 29.62		
≥80	90	97.57 ± 28.10		
Residence				
Urban	775	98.89 ± 21.10	*t* = 22.197	<0.001
Rural	344	63.35 ± 31.38		
Educational level				
Elementary school and below	165	66.87 ± 28.65	*F* = 32.028	<0.001
Middle school	353	88.09 ± 29.03		
High school or technical secondary school	331	91.52 ± 27.33		
College or bachelor degree	216	94.20 ± 26.67		
Master and above	54	104.83 ± 31.07		
Marital status				
Single	162	83.61 ± 35.63	*F* = 2.579	0.062
Married	894	89.18 ± 28.39		
Divorced	15	90.40 ± 12.70		
Widowed	48	79.19 ± 32.15		
Living status				
Living alone	204	82.51 ± 34.60	*F* = 6.890	<0.001
Living with spouse	594	87.59 ± 28.29		
Living with children	78	84.19 ± 29.73		
Living with spouse and children	243	94.65 ± 27.23		
Capita family monthly income (yuan)				
<1,000	99	82.06 ± 28.35	*F* = 8.037	<0.001
1,000–3,000	300	83.06 ± 26.40		
3,000–5,000	528	91.97 ± 29.52		
>5,000	192	87.66 ± 33.76		
Duration of diabetes (years)				
≤1	204	82.99 ± 29.91	*F* = 3.301	0.011
>1, ≤ 5	369	87.90 ± 29.83		
>5, ≤ 10	282	88.32 ± 30.22		
>10, ≤ 15	93	86.84 ± 27.78		
>15	171	94.04 ± 28.20		
Complication				
Yes	380	68.06 ± 33.66	*t* = −18.366	<0.001
No	739	98.20 ± 21.01		
Comorbidity				
Yes	460	74.89 ± 29.99	*t* = −13.245	<0.001
No	659	97.09 ± 25.76		
Treatment regimen				
None	288	82.74 ± 35.70	*F* = 5.085	<0.001
Oral hypoglycemic agent	513	90.98 ± 26.80		
Insulin	75	83.92 ± 25.10		
Oral hypoglycemic agent + insulin	195	91.40 ± 26.34		
Others	48	79.38 ± 32.27		

#### 3.2.2 The results of multiple linear regression analysis of the factors influencing physical activity in older adults with T2DM in the community

The factors that showed statistical significance in the univariate analysis results were considered independent variables, and multi-factor regression analysis was performed (α_in_ = 0.05, α_out_ = 0.10). The dependent variable was the total score of the change in physical activity for older adults with T2DM in the community. Table 8 displays the assignment of independent variables. According to the results of the multiple linear regression analysis, it was found that the factors such as residence, educational level, living status, complications and comorbidities were correlated with the overall score of the change in physical activity for older adults with T2DM in the community (*p* < 0.05). Great explanatory power (*R^2^* = 0.426, *ΔR^2^* = 0.424) and high overall significance (*F* = 165.514, *P*<0.001) are demonstrated by the regression models, suggesting that the identified factors have strong predictive power for changes in physical activity among older adults with T2DM in the community (Table 9).

**Table 8:** Variable assignments of factors.

Variable	Assignment
Age (years)	≥60, <70 = 1; ≥70, <80 = 2; ≥80 = 3
Residence	Urban = 1; Rural = 2
Educational level	Elementary school and below = 1; Middle school = 2; High school or technical secondary school = 3; College or bachelor degree = 4; Master and above = 5
Living status	Living alone = 1; Living with spouse = 2; Living with children = 3; Living with spouse and children = 4
Capita family monthly income (yuan)	<1,000 = 1; 1,000–3,000 = 2; 3,000–5,000 = 3; >5,000 = 4
Duration of diabetes (years)	≤1 = 1; >1, ≤ 5 = 2; >5, ≤ 10 = 3; >10, ≤ 15 = 4; >15 = 5
Complication	Yes = 0; No = 1
Comorbidity	Yes = 0; No = 1
Treatment regimen	None = 1; Oral hypoglycemic agent = 2; Insulin = 3; Oral hypoglycemic agent + insulin = 4; Others = 5

**Table 9:** Multiple linear stepwise regression analysis on influencing factors of physical activity in older adults with type 2 diabetes (*N* = 1,119).

Predicator	B	SE (B)	*β*	*t*	*P* value
Constant	98.073	3.841	-	25.533	<0.001
Residence	−26.181	1.618	−0.408	−16.178	<0.001
Educational level	1.700	0.665	0.063	2.558	0.011
Living status	1.581	0.675	0.054	2.342	0.09
Complication	14.255	1.673	0.228	8.523	<0.001
Comorbidity	11.005	1.512	0.183	7.277	<0.001

*R*^2^ = 0.426, Δ*R*^2^ = 0.424, *F* = 165.514, *p* < 0.001.

### 3.3 Mixed-method finding

After integrating and analyzing the qualitative and quantitative results, it was found that the two complemented, verified and expanded each other’s viewpoints in explaining the factors influencing physical activity among older adults with T2DM in the community (Table 10). Integrate the research results from two aspects: the MTM framework and beyond the MTM framework. (1) In the MTM framework, six topics have been identified, including 15 key factors. (2) Five topics were identified beyond the MTM framework, including eight key factors. Finally, 11 topics of this study were determined, including 23 key factors.

**Table 10:** Mixedmethods findings.

Theroy	Stage	Constructs	Qualitative study		Quantitative study	Mixed methods
			Thematic analysis	Topic modeling	Cross-sectional study	
MTM	Initiation (three primary constructs)	(1) Participatory dialogue	Benefits of moderate physical activity (physical health, mental health) The effects of Intense physical activity/excessive physical activity intensity	Focus on physical activity itself Perceive the benefits and disadvantages of physical activity	Not found	Focus on physical activity itself Benefits of moderate physical activity (physical health, mental health) The effects of Intense physical activity/excessive physical activity intensity
		(2) Behavioral confidence	Internal self-confidence	Not found	Not found	Internal self-confidence
		(3) Changes in the physical environment	Convenience and accessibility of fitness facilities Accessibility of health equipment (e.g., glucose meters) Accessibility and convenience of network health information resources	Fitness facilities in a physical environment	Residence	Convenience and accessibility of fitness facilities Accessibility of health equipment (e.g., glucose meters) Accessibility and convenience of network health information resources Residence
	Sustenance (three primary constructs)	(1) Emotional transformation	Shifts in disease acceptance	Emotion regulation types	Not found	Shifts in disease acceptance Emotion regulation types
		(2) Practice for change	The comprehensiveness of physical activity behavior types The persistence of physical activity	Other forms of physical activity other than the use of equipment	Not found	The comprehensiveness of physical activity behavior types The persistence of physical activity
		(3) Changes in the social environment	Social support from peers and family members	Social support for physical activity	Living status	Social support from peers and family members Being able to establish helpful social relationships Living status
			Being able to establish helpful social relationships			
Beyond MTM	Intrinsic disease		Comorbidity (e.g., myocardial infarction) Common health problems in older adults (e.g., backache, arthromeningitis)	Physical activity is limited by an intrinsic disease	Comorbidity	Comorbidity (e.g., myocardial infarction) Common health problems in older adults (e.g., backache, arthromeningitis)
	Stimulation		Stimulation	Not found	Not found	Stimulation
	Cultures		Educational level	Not found	Educational level	Educational level Traditional conception Family atmosphere
			Traditional conception			
			Family atmosphere			
	Uncontrollable factors		Not found	Uncontrollable factors that affect physical activity (e.g., weather, air quality, disease prevalence, interpersonal interactions, etc.)	Not found	Uncontrollable factors that affect physical activity (e.g., weather, air quality, disease prevalence, interpersonal interactions, etc.)
	Complication		Not found	Fear of complications caused by physical activity	Complication	Complication

## 4 Discussion

Utilizing a mixed-methods study, this study investigated the factors that influence changes in physical activity behavior in older adults with T2DM living in the community. We found eleven important elements related to the MTM constructs in qualitative study by manually coding the transcripts using thematic analysis. As supplements, we included three new factors, including six important factors beyond MTM. The semantic structure of the transcripts was also examined using the topic modeling approach LDA. As a result, we were able to identify five topics, including six important factors related to the MTM constructs, and supplement them with three new factors, including three important variables. Combining these two findings, we examined their similarities and differences and verified eleven topics, including twenty-one important factors, as the qualitative final outcomes. In the quantitative study results, we identified two key factors related to the MTM structure through cross-sectional surveys, and three supplementary factors were included. We compared and integrated the qualitative and quantitative study results, and finally determined 11 topics, including 23 key factors, as the set of the study results of this study.

The qualitative study included the opinions of the patients regarding the advantages and disadvantages of change in physical activity as well as the impact of changes in the physical environment on the changes in physical activity of older adults with T2DM in the community. Transforming physical environment that help initiate physical activity, so that these physical resources can more effectively promote the initiation of physical activity (64). Although patients during the interview paid close attention to the physical environment’s accessibility and convenience, most communities currently face issues like uneven and inadequately suited fitness facilities for older adults and poor hardware conditions. This aligns with Levinger’s research findings (65). Levinger discovered that the majority of the outdoor exercise equipment now available in the community is a copy of gym equipment (66). These equipment may not be suitable for all functional deficiencies related to aging, and thus may not be suitable for the older adults to improve balance and physical function. This makes older adults not inclined to choose community fitness equipment for exercise and physical activity. Consequently, it is vital to concentrate on the renovation of the old environment in the community, like installing fitness equipment suitable for older adults, installing elevators, optimizing the lighting system, installing electronic information dissemination equipment, and playing fitness videos suitable for older adults, such as Baduanjin and Tai chi. Through cross-sectional surveys, it was found that the residence (urban or rural) would also affect the changes in physical activity among older adults with T2DM in the community. This is owing to (1) the economic development lagging behind cities, rural areas have limited fitness place suitable for older adults, faultiness fitness equipment, the aging of equipment, and no one is in charge of the maintenance and repair of the equipment. Older adults also lack a correct understanding of physical activity (67). (2) Medical resources are scarce in rural areas. The recruitment of community public health service talents is insufficient. Medical staff lack reasonable training. The coverage rate of health education is low. Older adults with T2DM in rural areas have insufficient understanding of the importance of physical activity in blood sugar control and lack professional guidance for scientific and effective physical activity (68). (3) In rural areas, many family members go out to work, and older adults lack accompany and supervision, making it difficult for them to maintain regular physical activity. Furthermore, there are relatively few health activities organized by communities, and there is a lack of incentives for collective physical activity (69). Therefore, it is necessary to actively carry out physical activity for older adults with T2DM in rural communities, strengthen publicity on the prevention and treatment of chronic diseases and correct physical activity among older adults in rural communities, encourage professional exercise physiologists to go deep into rural areas, and take multiple measures to expand sports and fitness place for older adults in rural areas (70).

The study focuses on internal self-confidence in relation to behavioral confidence. The majority of responders said they were very confident in their ability to keep up their physical activity and blood sugar levels, both now and in the future. However, research indicates that older adults with T2DM disregard their health demands, neglect blood glucose management, and no hope for blood glucose control because they lack health knowledge and a low sense of self-worth (19). The findings of our research are inconsistent with this, and the precise causes require additional confirmation. In the outcome analysis of emotional transformation, the study examines how patients control their emotions to encourage both the initiation and sustenance of physical activity in addition to accepting the illness across its whole duration. Nevertheless there are some stimulations such as fall down. As older adults grow older, the bones of older adults may develop osteoporosis, with decreased bone density and weakened bone strength, making the bones more fragile (71). This can cause patients to be afraid of fractures or paralysis caused by falls, which can easily induce mood swings and impair their internal confidence in continuing physical activity (72). Furthermore, Carr et al. (73) discovered that older adults’ family and society always give their blood glucose more attention than their mental health, and that support may gradually deteriorate as their impairment worsens. These findings have an impact on how older adults with T2DM alter their health-related behaviors in the community. Therefore, community health care providers should pay attention to the psychological and emotional problems during the process of physical activity behavior changes in older adults with T2DM not only in the early stage of the disease but also throughout the course of the disease. They should also remind patients to formulate appropriate activity plans based on the assessment of their own conditions when doing physical activity, to be moderate and appropriate, and to prevent falls or other injury incidents.

In the result analysis of practice for change, this study not only focused on the comprehensiveness and persistence of physical activity, but also on active reflection and post-reflection behavioral practices. As the foundation of comprehensive diabetes care, blood glucose monitoring is essential for managing and treating diabetes while also serving as a basis for adjusting diet, physical activity, and insulin dosage (74–76). Through the identification minor health issues, blood glucose monitoring enables patients to comprehend the trends, fluctuations, and influencing variables of blood glucose changes, leading to more thorough and efficient diabetes care (77).

Some participants have glucometers at home. And they will promptly adjust their physical activity plans according to the changes in their blood sugar levels. Furthermore, to avoid hypoglycemia, they suggest preparing some glucose or sugar cubes before physical activity. However, some participants similarly disregard the significance of blood glucose monitoring. This is because blood glucose monitoring compliance may be impacted by a number of external factors, including patients’ fear of pain, a lack of understanding of the significance of blood glucose monitoring, negative emotions brought on by abnormal blood glucose values, and a reluctance to take the initiative to self-monitor blood glucose (78, 79). This is consistent with Zhou Yan et al.’s research findings (80), which indicate that diabetic patients at home as well as abroad have a shared dread of blood glucose monitoring, which causes them to be reluctant or neglect to obtain it when necessary. With the benefits of security, efficiency, noninvasion, and continuity, the dynamic glucose meter can track blood glucose levels in real time (81). The objective blood glucose data can also serve as a basis for modifying patients’ physical activity and other health-related behaviors.

Patients reported greater support from friends, family, and other patients when the long-term effects of the changes in the physical environment on physical activity were examined. Although they are a significant influence supporting changes in the physical environment, professionals like community health education nurses and health education specialists are not as often emphasized (82). In contrast to community health care systems abroad, China’s community health services were introduced later, are still in early stages, and have a lack of consistency (83). The inability of community health service providers to address the health issues of every patient in the community is caused by a lack of human resources and an inadequate ratio of community health service providers to the residents. Members of community health services also typically have poor professional qualifications and lack professional nursing and medical services (84). In order to give residents quick access to health advice, community health service centers can create an information digital health platform by integrating various health resources and health science expertise (85). It is distinguished by “connecting health care with other social resources” and creates a multi-level “policy-health system-community-individual” collaborative network (86, 87). Encourage senior medical students to volunteer for community health service projects and strengthen the linkages between hospitals, communities, and schools (88). To address the lack of qualified staff in community health services, hospital medical professionals should routinely provide free medical care in the neighborhood (89). Furthermore, community health service providers might be encouraged to receive frequent knowledge and guidance from hospital exercise physiologists and professional medical staff to improve their professional qualities (84).

We find five additional topics in beyond MTM. Different educational levels in the cultural field provide different perspectives on how physical activity behavior changes. Patients’ behavioral patterns and health conceptions are somewhat shaped by traditional concepts, which might help or hinder the development of physical activity. From the perspective of dyadic coping, we may learn how the attitudes and behaviors of patients and their spouses regarding the illness affect changes in physical activity within the family atmosphere (90). In addition, there are four important topics: *Stimulation, Intrinsic disease, Complication* and *Uncontrollable factors*. Stimulation primarily focuses on how sudden events brought on by physical activity (like falling) affect patients’ physical activity changes. This includes both internal and external warning effects, which can influencethe awareness of physical activity in older adults with T2DM. During the research process, some participants’ concerns were not only concerning T2DM but also regarding comorbidities (like myocardial infarction, cataracts) and common health problems in older adults (such as lumbago, synovitis). This was discovered when examining the effect of intrinsic disease in older adults on physical activity. Research has demonstrated that comorbidity not only complicates diabetes diagnosis and treatment, but also exposes patients to a situation where they must take numerous medications, which significantly raises the disease burden of diabetic patients and readily results in a decline in medication compliance (91, 92). In qualitative interviews, we also found that the emergence of common health problems among older adults would limit the activity level of older adults with T2DM in the community, thereby affecting the initiation and sustenance of physical activity changes. Therefore, by providing health education on related diseases, community health service providers can help older adults with T2DM who have comorbidities or common health problems better understand the occurrence, development, and prevention strategies of these diseases, as well as improve their confidence and self-management ability (93). Furthermore, based on the patients’ own conditions, personalized physical activity plans are designated for them, thereby indirectly enhancing the patients’ confidence in physical activity. Complications mainly involve the impact of complications caused by T2DM on patients, such as refusing physical activity for fear of hypoglycemia. The key to uncontrollable factors lies in the irresistible external factors that affect the patient’s physical activities, such as weather conditions, air quality, disease prevalence, interpersonal communication. Therefore, community healthcare workers should formulate flexible and alternative physical activity prescriptions in combination with individual health conditions and different external environmental conditions.

In addition, this study still has some limitations. First off, this study might not be applicable to older adults in different nations or those from various socialdemographic characteristics or cultural backgrounds due to its potential geographic limitations. Hence, our findings must be verified in in different geographic regions and cultures. Additionally, using a mixed-methods study, we applied the MTM to analyze the factors influencing the change in physical activity of older adults with T2DM in the community. However, we are uncertain if these factors are suitable for children and hospitalized patients with diabetes. Lastly, the results of the qualitative research have not been confirmed in an independent population, and the sample size is small. Although we determined the sample size and received additional factors besides this theory in accordance with the principle of theoretical information saturation. Nonetheless, from the standpoint of theoretical innovation and the depth of the research, it is necessary to include more participants, including increasing the sample size and introducing groups from different regions to ensure the comprehensiveness and representativeness of the study results. As an alternative, validation in a different cohort would improve the results’ generalizability and dependability.

## 5 Conclusion

The *Chinese diabetes behavior and lifestyle intervention guidelines (2024)* recommend incorporating behavioral change theories into intervention strategies and emphasize that intervention measures guided by behavioral change theories or their components can more effectively modify various health behaviors and diabetes management. For the first time, we adopted the MTM as a theoretical framework to deeply analyze the specific factors influencing physical activity in older adults with T2DM, laying the foundation for implementing precise interventions guided by behavioral change theory. By integrating qualitative and quantitative study results, we obtained mixed-methods study findings, including 15 factors within the MTM theoretical framework and 8 factors beyond MTM, such as “Focus on physical activity itself,” “The persistence of physical activity,” and “Traditional conception.” These factors were not mentioned in our previous systematic literature search. These new discoveries provide a strong theoretical framework and empirical support for creating more individualized and scientific strategies for improving physical activity behaviors, and offers a new perspective for the next step of constructing physical activity programs for older adults with T2DM in the community.

## Data Availability

The original contributions presented in the study are included in the article/supplementary material, further inquiries can be directed to the corresponding authors.
