# Correction: From the perspective of multi-theory model, factors influencing physical activity among community-dwelling older adults with type 2 diabetes in China: a mixed-methods study

**DOI:** 10.3389/fpubh.2025.1713194

**Published:** 2025-10-23

**Authors:** Panpan Huai, Bo Zhang, Jingjing Sun, Rui Xu, Linghui Zhang, Xiao Qiao, Weili Sun, Hui Yang, Jinli Guo, Huancheng Su

**Affiliations:** ^1^School of Nursing, Shanxi Medical University, Taiyuan, China; ^2^Balingqiao Community Health Service Center of Xinghualing District, Taiyuan, China; ^3^Shanxi Bethune Hospital, Shanxi Academy of Medical Sciences, The Third Hospital of Shanxi Medical University, Tongji Shanxi Hospital, Taiyuan, China; ^4^The First Clinical Medical College of Shanxi Medical University, Taiyuan, China; ^5^The Second Clinical Medical College of Shanxi Medical University, Taiyuan, China

**Keywords:** multi-theory model, type 2 diabetes, older adult, community, factors, mixed-method study

There was a mistake in Figure 1 as published. In the measure tool of the Quantitative study, the “(2) the Measuring Change in Physical Activity Questionnaire” was not included. The corrected Figure 1 appears below.

**Figure 1:**
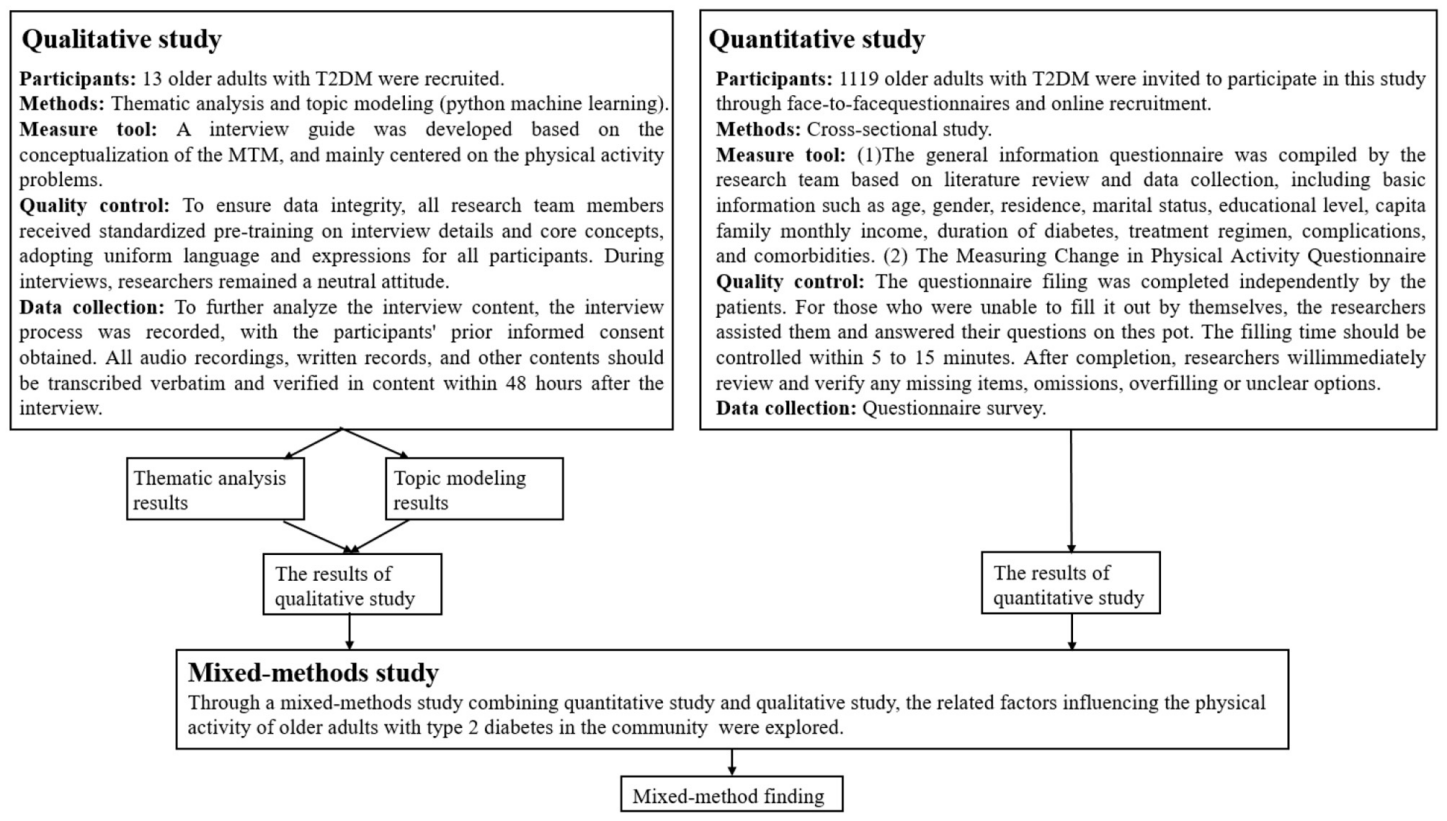
Design of the study using a mixed method of quantitative study and qualitative study.

Figure 1. Design of the study using a mixed method of quantitative study and qualitative study.

We found that in 2.3.2 Measures, there is a lack of an introduction to “Measuring Change in Physical Activity Questionnaire.”

A correction has been made to the section 2.3.2 Measures as follows:

## 2.3.2.2 Measuring change in physical activity questionnaire

Intentions to engage in physical activity were evaluated using the Measuring Change in Physical Activity Questionnaire (MCPAQ). It was originally developed in English based on the MTM construct by Sharma (62). The higher the scores of changes in physical activity, the greater the likelihood of conducting physical activity behavior change. Yang et al. (63) obtained authorization from the original authors of the MCPAQ and conducted a cross-cultural adaptation to develop a Chinese version of the scale. This version was validated in hypertensive patients and demonstrated good reliability and validity: Cronbach's alpha was 0.911 for the overall scale. The scale is considered broadly applicable across diverse populations.

The original version of this article has been updated.
